# Fragrance-Free Policies: A Scoping Review of Definitions, Implementation, and Gaps

**DOI:** 10.3390/ijerph23081089

**Published:** 2026-08-21

**Authors:** Sudipta Roy, John Molot, Robert Lattanzio, Jennifer Armstrong, Riina Bray, Adrianna Trifunovski, Rohini Peris

**Affiliations:** 1 Association Pour la Santé Environnementale du Québec-Environmental Health Association of Québec (ASEQ-EHAQ), Saint-Sauveur, QC J0R 1R1, Canada; sudipta@aseq-ehaq.ca (S.R.); adrianna@aseq-ehaq.ca (A.T.); 2 Faculty of Medicine, University of Ottawa, Ottawa, ON K1H 8M5, Canada; jmolot@rogers.com; 3 ARCH Disability Law Centre, Toronto, ON M5J 2H7, Canada; robert.lattanzio@arch.clcj.ca; 4 Ottawa Environmental Health Clinic, Ottawa, ON K2H 5A8, Canada; dr@oehc.ca; 5 Environmental Health Clinic, Women’s College Hospital, Toronto, ON M5S 1B2, Canada; riina.bray@rogers.com; 6 Department of Family and Community Medicine, University of Toronto, Toronto, ON M5G 1V7, Canada

**Keywords:** indoor air quality, multiple chemical sensitivity, volatile organic compounds, fragrance-free policy, environmental health policy, accessibility

## Abstract

**Highlights:**

**Public health relevance—How does this work relate to a public health issue?**

- Fragrance-free policies are increasingly adopted to address indoor air quality concerns and reduce fragrance-related exposure.
- This scoping review identified substantial variability in the design, implementation, and enforcement of fragrance-free policies across sectors.

**Public health significance—Why is this work of significance to public health?**

- First comprehensive synthesis of fragrance-free policy characteristics across workplace, healthcare, educational, and public settings.
- Findings highlight key governance gaps, limiting the effectiveness of fragrance-free policies as environmental health interventions.

**Public health implications—What are the key implications or messages for practitioners, policy makers and/or researchers in public health?**

- Our findings offer evidence-informed recommendations for strengthening policy effectiveness and accessibility.
- Clear guidance, education, and accountability mechanisms can enhance policy compliance and promote more inclusive indoor environments.

**Abstract:**

Background: Fragrance-free policies are increasingly adopted to improve indoor air quality and reduce fragrance exposure linked to adverse health outcomes and accessibility barriers. This scoping review mapped the evidence on fragrance-free policies across sectors. Methods: Following Arksey and O’Malley’s framework and PRISMA-ScR guidelines, we searched five peer-reviewed databases and grey literature sources. Documents describing formal scent-free or fragrance-free policies or guidance in workplaces, healthcare, educational, or public settings were included. Data were charted on policy characteristics, enforcement mechanisms, implementation supports, and governance features. Results: Sixty-three documents were included. Findings revealed substantial variability in terminology, scope, and enforcement. Many policies relied on voluntary compliance and awareness-based strategies, with limited integration of structural supports such as procurement controls, staff training, and evaluation frameworks. Enforcement mechanisms were predominantly reactive, responsibilities were inconsistently defined, and consequences for non-compliance were limited. Although many policies referenced multiple chemical sensitivity or fragrance sensitivity, accommodation pathways and accountability structures were frequently lacking. Four key themes indicate that many fragrance-free policies function as accommodation tools rather than integrated environmental health interventions. Conclusions: Strengthening definitional clarity, institutional accountability, and structural integration may enhance effectiveness and support more consistent, equitable, and evidence-informed approaches to reducing fragrance-related exposures in indoor environments.

## 1. Introduction

Indoor air quality (IAQ) has emerged as a major environmental health concern and an important determinant of population health, occupational safety, and environmental equity. Because people spend approximately 90% of their time indoors, exposure to indoor pollutants can contribute substantially to overall environmental exposures [1]. Concentrations of volatile organic compounds (VOCs) and other chemical contaminants are often higher indoors than outdoors and may, in some circumstances, reach levels up to ten times greater. Growing evidence indicates that indoor environmental conditions can influence health, well-being, and disease risk, particularly among vulnerable populations [1,2,3,4]. Indoor environments contain complex mixtures of biological, chemical, and physical contaminants arising from building materials, cleaning agents, furnishings, combustion sources, and consumer products that influence human exposures and health [5]. Among these, fragranced consumer products represent a pervasive and comparatively under-regulated source of indoor chemical exposure. Scientific research has demonstrated that fragranced consumer products emit a diverse array of VOCs, including terpenes such as limonene, as well as aldehydes and other reactive compounds, many of which are classified as toxic or hazardous by federal laws and are not disclosed on product labels, also contributing to indoor VOC burdens [6,7,8,9]. Many VOCs emitted from fragranced products can activate transient receptor potential (TRP) channels, such as TRPA1 and TRPV1, on sensory nerves in the airways, which can cause irritation, inflammation, and pain without involving an allergic response [10,11]. These mechanisms provide a biological explanation for symptoms linked to fragrance exposure [12].

For the purposes of this review, the terms “fragrance-free” and “scent-free” are used interchangeably, unless otherwise specified. References to fragrance-free policies therefore include policies labelled as scent-free, fragrance-reduced, or similar terms across jurisdictions.

### 1.1. Health and Societal Effects of Fragranced Products

Fragranced consumer products (or fragranced products) are products containing intentionally added fragrance ingredients to impart aroma or scent [9,13]. These include commonly used items such as air fresheners, disinfectants, laundry detergents, fabric softeners, scented candles, essential oils, and personal care products [14]. Such products are widely used across residential, occupational, healthcare, educational, and commercial settings. Although fragrance ingredients are evaluated under industry-established safety standards [15], research has linked fragranced consumer products to adverse impacts on IAQ and human health. Additionally, newly introduced products, materials, and furnishings, including carpeting, synthetic textiles, clothing, furniture, paint, and recently painted buildings, can emit VOCs and odours that may contribute to adverse health effects in sensitive individuals [16,17].

Epidemiological and population-based research has documented self-reported adverse health and societal effects associated with fragranced products among the general population. Reported adverse health outcomes include headaches and migraines [18,19,20,21], asthma and asthma exacerbations [21,22,23,24,25], mucosal irritation [21,26], dermatologic reactions [21,27,28,29], and photosensitivity reactions, including phototoxicity and photoallergy [30]. Occupational health surveillance has similarly implicated fragrance exposure in work-related asthma cases [31].

Recent non-target screening studies have demonstrated strong genotoxic activity in compounds present in perfumes [32,33]. Certain fragrance ingredients have also been characterized as neurotoxic [34,35], and some substances, including synthetic musks and Lilial, have been identified as potential endocrine disruptors [23,36,37], with Lilial additionally associated with potential adverse effects on fertility [37]. However, the long-term health implications of chronic lifelong exposure remain uncertain.

Health impacts have been documented among vulnerable populations, including individuals with autism, asthma, and multiple chemical sensitivity (MCS) [22,24,38,39]. In some populations, the severity of reactions may be disabling [21]. Fragrance exposure is frequently involuntary, often described as “second-hand scent”—occurring through proximity to others’ product use in shared indoor spaces [14]. Such exposure may create barriers to participation in workplaces, healthcare facilities, hotels, and other public and social environments. Surveys indicate avoidance behaviours, lost workdays and job loss associated with fragrance exposure [13,40,41,42,43]. Emerging research has found that fragrance exposure also causes significant impacts on life, including physical breakdown (collapse, fainting), social isolation, and reduced quality of life [42,43,44].

### 1.2. Prevalence of Fragrance Sensitivity and MCS

Fragrance sensitivity refers to adverse health effects triggered by exposure to fragranced consumer products [21,22]. The American Medical Association (AMA) Council on Science and Public Health has examined fragrance sensitivity within overlapping diagnostic constructs, including MCS, chemical sensitivity, chemical intolerance, toxicant-induced loss of tolerance (TILT), and related terminology [45]. The coexistence of multiple labels reflects variability in clinical and policy approaches to describing fragrance- and chemical-related health effects. For clarity and consistency, this review uses the terms MCS and fragrance sensitivity when referring to fragrance-related health impacts.

Epidemiological data indicate that fragrance-related health effects are not uncommon. Approximately 1.9% of the general population across five European countries (Germany, Italy, the Netherlands, Portugal, and Sweden) has a fragrance contact allergy [46]. In the United States, nearly one-third of adults report irritation from fragranced products used by others, highlighting the phenomenon of “second-hand scent” exposure [22]. An international survey across the United States, Australia, the United Kingdom, and Sweden found that approximately 32.2% of adults reported health effects associated with fragranced product exposure [21].

In Canada, data from the Canadian Community Healthy Survey (CCHS) indicate that approximately 1.13 million Canadians (≈3.5%) reported a diagnosis of MCS in 2020 [47]. Preliminary 2025 CCHS data (January–July) indicate that over 3.1 million Canadian adults (9.4%) report having MCS, while approximately 2.7% report having received a diagnosis [48]. This discrepancy suggests persistent gaps in clinical recognition, diagnosis, and access to care. Sex-disaggregated estimates from the 2025 CCHS further indicate that women are disproportionately affected; approximately 12.8% of women report having MCS, equivalent to roughly 1 in 8 women. Consistent with prior analyses, approximately 72% of individuals reporting diagnosed MCS were women [47].

Evidence from other settings similarly indicates that women experience higher rates of fragrance-related sensitivities, with some estimates suggesting rates approximately twice those observed in men [46]. This disproportionate distribution may reflect differences in exposure patterns, including higher use of fragranced cosmetics, skincare products, and perfumes, alongside potential biological and sociocultural factors. It may also possibly be due to the fact that estrogen hormones can stimulate, upregulate, and sensitize chemosensitive TRPA1 and possibly TRPV1 receptors, which are sensitized in MCS [12]. Variability in prevalence estimates across studies reflects ongoing differences in disease definitions (e.g., MCS, chemical sensitivity, chemical intolerance, environmental illness, TILT, and fragrance sensitivity) as well as measurement approaches, including self-reported conditions versus clinically confirmed diagnoses [45]. Given that fragrance intolerance is commonly reported among individuals with MCS and may extend beyond formally diagnosed cases, these figures indicate substantial population-level relevance. Comparable patterns of fragrance-related adverse reactions have been reported internationally; for example, a study in Saudi Arabia found that 50.6% of participants experienced at least one adverse reaction to cosmetic products, including perfumes [49].

### 1.3. Fragrance-Free Policies

Affecting millions across diverse age groups and regions, fragrance sensitivity has emerged as a significant public health concern. Surveys indicate that at least twice as many people prefer fragrance-free environments in workplaces, healthcare facilities, and public spaces, with even stronger preferences among vulnerable sub-populations and individuals without fragrance sensitivity [14,21,38,42]. Fragrance-free environments promote substantive disability inclusion by reducing environmental barriers that can trigger or exacerbate symptoms for individuals living with MCS, asthma, and other chronic health conditions, thereby supporting equitable participation in workplaces and public spaces [50,51]. In response, fragrance-free policies have emerged as institutional strategies to reduce indoor exposures [52].

Fragrance-free policies are formal protocols or guidelines implemented by public or private organizations to promote environments free from fragranced and related chemical products [14]. These policies typically prohibit fragranced items and require the use of the lowest-VOC and least-toxic products to reduce unnecessary chemical emissions indoors. They apply not only to personal scents but also building-related products such as cleaning agents, laundry detergents, air fresheners, and other fragranced consumer or institutional products. Some policies further address additional preventable airborne exposures, including masking agents, pesticides, smoke, and non-odorous irritants that can affect IAQ.

Similar to other workplace health and safety measures, such as anti-harassment policies, fragrance-free policies apply to all employees and are intended to protect the health, safety, and well-being of individuals with MCS [53]. They have been implemented across healthcare systems, universities, government agencies, schools, libraries, and private workplaces in North America, Europe, and Australia to accommodate individuals affected by fragrances, including those with MCS [14,53]. Despite their increasing adoption, fragrance-free policies demonstrate substantial variability in terminology, scope, enforcement, and accommodation provisions. Some are framed as voluntary awareness initiatives, whereas others adopt mandatory language with formal enforcement procedures. To date, policy analyses have largely been descriptive and geographically limited, with insufficient synthesis of global guidance, enforcement mechanisms, accommodation frameworks or pathways, and implementation structures across jurisdictions.

### 1.4. Objective

The objective of this scoping review was to map the breadth of evidence on fragrance-free policies, including how they are defined, the populations to whom they apply, their level of enforceability, accommodation provisions for individuals with MCS, and key implementation gaps across sectors. By synthesizing evidence across sectors and jurisdictions, this review aimed to inform the development of more consistent and effective environmental health policies that fully protect vulnerable populations.

## 2. Materials and Methods

### 2.1. Study Design

We conducted this scoping review as part of a broader IAQ research study examining fragrance exposure governance and environmental accessibility. We followed Arksey and O’Malley’s framework for identifying, selecting, and synthesizing evidence [54] and PRISMA-ScR reporting guidelines (File S1) to ensure methodological transparency and reproducibility [55]. Given the heterogeneity of policy documents and substantial contribution of grey literature, a scoping review is an appropriate methodology to map the breadth of available evidence, clarify key policy features, and identify gaps in implementation and research.

### 2.2. Search Strategy

A comprehensive search strategy was used to identify both peer-reviewed and grey literature on fragrance-free policies. Electronic database searches were conducted in PubMed, MEDLINE, ScienceDirect, Web of Science, and CINAHL. To capture policy documents and implementation guidance, grey literature searches were conducted across websites of government and public health agencies, healthcare institutions, accessibility bodies, educational institutions, and advocacy or professional organizations. Websites were searched using their internal search function where available, supplemented by manual browsing of relevant policy and accessibility webpages and targeted keyword searches (e.g., fragrance-free, scent-free, policy, guideline, and related terms). Reference lists of included sources were also screened to identify additional relevant materials. Key search terms were identified through consultations between the author and research team members, which included combinations of keywords related to fragrance-free policies and indoor environments (e.g., workplace, education, and public spaces). Boolean operators were used to connect subject headings and keywords. There was no restriction on publication type or date. The final searches were conducted in March 2026.

### 2.3. Eligibility Criteria

Documents were included if they contained explicit scent-free or fragrance-free policies or formal guidance; applied to healthcare settings, workplaces, educational institutions, or other public spaces; were available in English or French; and addressed elements of implementation, compliance, or accommodation. Materials were excluded if they consisted solely of opinion without policy relevance, were product marketing materials, or were unrelated to environmental exposure policy.

### 2.4. Study Selection

All records identified through databases and grey literature searches were imported into Covidence. Duplicates were automatically removed prior to screening. Although peer-reviewed literature was searched, no journal articles met the inclusion criteria. Peer-reviewed publications addressing fragrance-free policies were largely descriptive commentary or advocacy-oriented analyses rather than formal institutional policy documents and therefore did not satisfy inclusion requirements. Because the objective of our scoping review was to characterize existing fragrance-free policies, only documents containing explicit policy statements or implementation guidance were eligible. In practice, fragrance-free policies are primarily developed and disseminated by governments, healthcare organizations, educational institutions, and professional organizations rather than published in peer-reviewed journals. Consequently, this scoping review draws exclusively on grey literature, including government, institutional, and professional policy documents, which represent the principal and most appropriate source of evidence, providing direct access to policy documents used in real-world settings. Title and abstract screening were conducted by the primary author (SR), with a subset (half of records) independently reviewed by a second researcher (AT) to verify eligibility decisions. Discrepancies were resolved through discussion with a third reviewer (RP) until consensus was reached.

### 2.5. Data Extraction

For each included document, detailed information was extracted using a structured data charting form developed specifically for this review. The data charting form was implemented in Microsoft Excel. The form was designed to systematically capture policy characteristics and ensure consistency across diverse sources. Data were systematically extracted on key descriptive and implementation variables, including the policy title, issuing organization, and setting. Policy characteristics captured included policy status, definitions, existence of a default fragrance-free standard, applicability to visitors and contractors, explicit reference to MCS, specified complaint, and enforcement mechanisms, including any consequences for non-compliance. Implementation and accountability elements included burden of proof, cultural exemptions, supply-chain controls, inclusion of facilities or maintenance provisions, and a stated policy trigger or rationale. Together, these variables enabled a comprehensive assessment of policy structure, implementation supports, and governance features.

To support consistent classification, the research team iteratively developed a coding framework based on an initial review of included documents and relevant policy analysis literature. Policy status categories were operationalized using predefined criteria: mandatory policies reflected enforceable requirements; advisory and voluntary policies indicated non-binding guidance; guidelines represented formal but non-enforceable recommendations; and awareness-based materials captured informational or educational resources without institutional directives. To distinguish different types of records included in this review, policy tiers were operationalized into five categories based on their purpose, level of authority, and predominant policy characteristics: Tier 1 included enforceable institutional policies with mandatory language, Tier 2 included advisory institutional policies, Tier 3 included government directives or accessibility plans, Tier 4 included awareness campaigns such as posters or educational materials, and Tier 5 included implementation toolkits, templates, or advocacy resources. Documents containing elements spanning multiple tiers were classified based on the dominant policy approach, with mandatory and enforceable policy elements taking precedence over educational or awareness-oriented components. Burden of proof classifications were assigned based on whether responsibility for compliance or accommodation rested with the employer (default fragrance-free environment or proactive restriction) or individual (requiring accommodation requests, documentation, or complaint triggers), or if it was unclear (encouragement-based or non-specific language). Enforcement mechanisms were interpreted based on the presence of formal processes, including complaint procedures, monitoring, or consequences such as progressive discipline, human resources review, or occupational health and safety processes.

Data extraction and coding were conducted using a two-stage review process, with a subset of documents independently reviewed by a second reviewer to ensure reliability and verify consistency in classification. Any ambiguities or discrepancies in classification were resolved through discussion and consensus among the research team to minimize subjectivity and ensure methodological rigor.

## 3. Results

**Figure 1:**
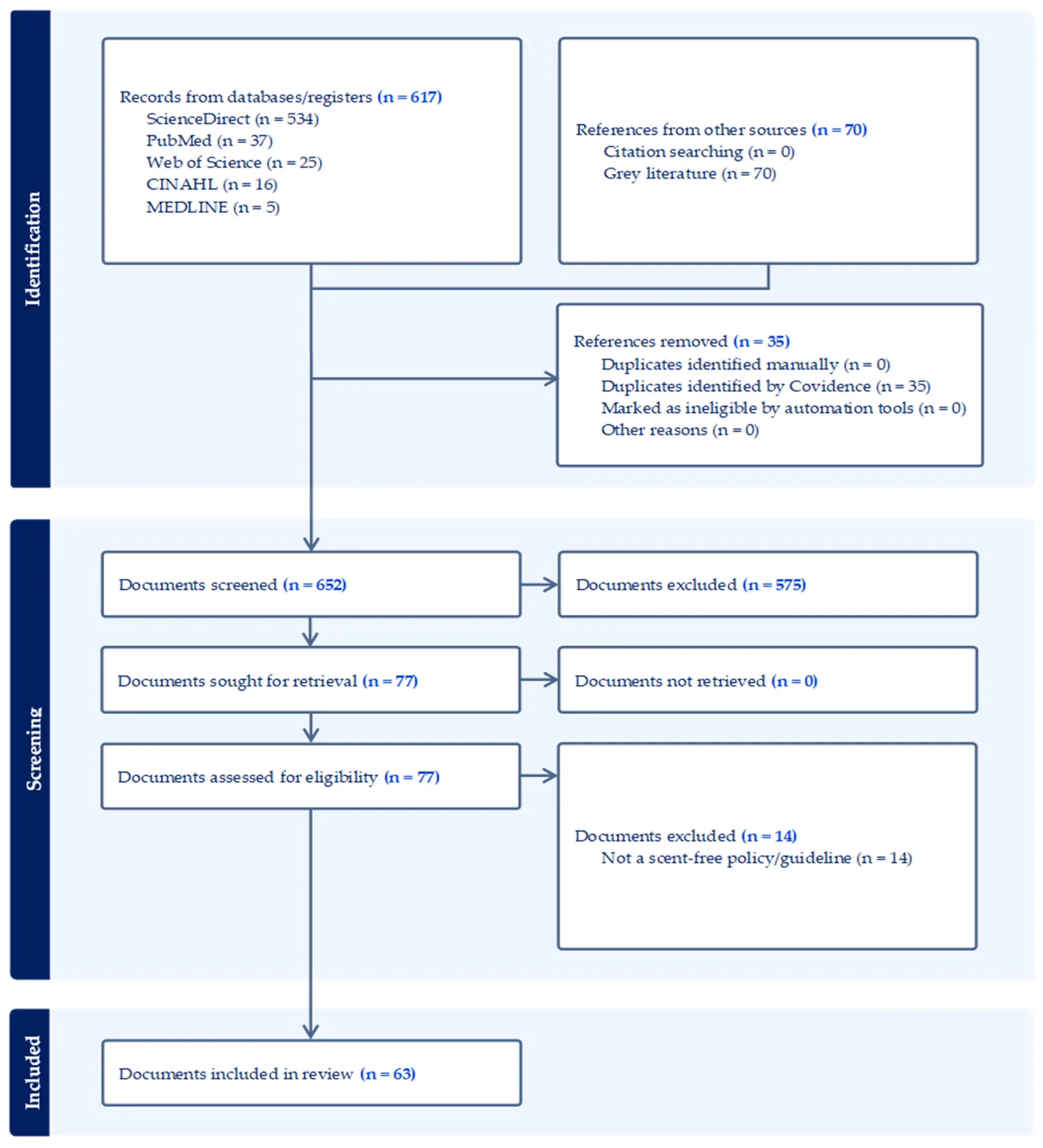
PRISMA-ScR flow diagram.

The search identified 687 potentially relevant records across databases and grey literature sources. Of the records identified, 617 were retrieved from peer-reviewed databases and 70 from grey literature sources. After removal of duplicates, 575 records were excluded based on title and abstract screening, leaving a total of 77 records for full-text screening. Of these, 63 documents were included for the final synthesis. The selection process is presented in the PRISMA-ScR flow diagram (Figure 1). Document types included formal policies, guidelines, institutional protocols, and implementation frameworks.

The characteristics of the included policies are presented in Table S1. Most of the documents were from Canada and the USA (n = 59), with additional examples from other international jurisdictions. However, the included records represented a diverse range of sectors, including healthcare organizations, government agencies, workplaces, educational institutions, and public facilities. Most policies (n = 49) explicitly applied to employees and staff, particularly within workplace and healthcare settings. Based on policy tier classification, healthcare and governmental settings most frequently adopted mandatory policies (n = 12) using directive language, whereas advisory or voluntary policies, framed as recommendations or awareness-based approaches, were more common in educational and community settings. Explicit reference to MCS or fragrance sensitivity within occupational health, disability, or accessibility frameworks was present in more than half of the documents (n = 48), and the majority applied to visitors as well as staff, indicating recognition of shared indoor environments.

Table S2 summarizes the substantive policy elements. Explicit operational definitions were provided in 30 (47.6%) documents, while others did not clearly specify which products were included or what criteria defined compliance. Several policies established a default expectation of minimizing fragrance exposure; however, a default fragrance-free standard was established only in 23 policies (36.5%). Across 63 documents reviewed, a formal complaint mechanism was specified in 37 policies (58.7%), and 41 policies (65.1%) described at least one enforcement mechanism for addressing non-compliance, which commonly involved HR review, occupational health and safety processes, or supervisory/managerial intervention. However, explicit consequences for non-compliance were identified in only 14 policies (22.2%), suggesting that enforcement structures were often informal or educational rather than disciplinary. The allocation of burden of proof also varied, with 11 policies (17.5%) requiring individuals to substantiate fragrance-related harm to trigger action. In 19 policies (30.2%), the employer/organization was primarily responsible for maintaining a low-exposure environment, and these policies frequently used directive language. The allocation of responsibility remained unclear in 27 documents (42.9%). While procedural elements were present in many policies, the allocation of responsibility was often unclear.

Table S3 summarizes the implementation supports and governance features. In 36 policies (57.1%), signage requirements were identified, while formal staff training was referenced in only 10 policies (15.9%). These findings reflect moderate reliance on environmental prompts and limited emphasis on systematic education to encourage compliance. Structural supports, including supply-chain or procurement controls (29 policies, 46.0%) and integration with facilities management (30 policies, 47.6%), were inconsistently incorporated, indicating fewer policies addressed institutional sources of fragrance exposure. Only two policies (3.2%) described explicit cultural exemptions, and four policies (6.3%) specified medical documentation requirements, highlighting the limited presence of clearly defined procedures for individualized accommodation. Although a responsible authority was identified in 47 policies (74.6%), review cycles were specified in only 14 policies (22.2%), indicating limited attention to formal monitoring or evaluation frameworks. A rationale or policy trigger was reported in 60 policies (95.2%), indicating that most policies articulated a justification for implementation.

Although procedural elements were present in many policies, responsibility and accountability structures were frequently ambiguous. Across policy tiers, several structural patterns emerged. Tier 1 policies were more consistently associated with institutional responsibility and the presence of structural implementation elements, including default fragrance-free standards, enforcement mechanisms, and supply-chain or facilities-related controls. By contrast, Tier 4 awareness materials generally lacked complaint, enforcement, and accountability structures, reflecting their informational rather than operational function. Tier 5 documents reflected a more mixed position: although many included complaint or enforcement language, they less often incorporated procurement controls, facilities integration, or other formal governance supports. Policies with advisory or voluntary status were more likely to rely on complaint-based mechanisms and non-binding language, and were frequently associated with unclear burden of proof classifications. Together, these patterns suggest that stronger policy status was associated not only with directive language but also with greater structural integration and clearer institutional accountability.

### 3.1. Thematic Synthesis

Across the 63 documents reviewed, four overarching themes emerged, as presented in Section 3.1.1, Section 3.1.2, Section 3.1.3 and Section 3.1.4.

#### 3.1.1. Conceptual Variability

Thematic analysis revealed substantial heterogeneity in how fragrance-free policies were conceptualized and framed across institutions. Policies were described using terms such as “scent-free”, “fragrance-free”, “scent-reduced”, and “fragrance-aware”; however, these terms were used inconsistently and did not necessarily represent equivalent policy approaches. This absence of standardized definitions highlights considerable conceptual variability within the field. Several policies referred broadly to “fragranced personal care products” without listing specific examples (e.g., perfumes, lotions, cleaning agents, laundry products, or air fresheners), which created ambiguity regarding expectations and enforcement thresholds. Most policies articulated a general objective to minimize or eliminate exposure; however, the scope and regulatory strength varied considerably. Inclusion of visitors was inconsistent and often framed as voluntary or advisory rather than mandatory, suggesting differential expectations between staff and the public. Policy status further reflected conceptual differences, ranging from enforceable institutional directives to voluntary awareness campaigns. There were inconsistencies across documents regarding explicit recognition of MCS or fragrance sensitivity as a rationale for implementation. Policies that referenced MCS were more likely to frame fragrance-free measures as accessibility or occupational health obligations, whereas those without such reference tended to position fragrance reduction as a matter of courtesy or personal preference. This variability in definitions, regulatory framing, and recognition of MCS suggests decentralized policy development and inconsistent conceptualization of fragrance exposure as a health or behavioural issue. From a governance perspective, such fragmentation may limit enforceability, reduce institutional coherence, and weaken the legitimacy of fragrance-free initiatives as environmental health interventions.

A recent medical policy review further illustrates this diagnostic heterogeneity. The AMA’s 2025 examination of fragrance sensitivity referenced multiple related frameworks, reflecting the continued coexistence of overlapping clinical constructs [45]. This multiplicity of terminology may contribute to fragmented governance approaches, as institutions develop fragrance-free policies without standardized definitions or consistent reference to underlying health frameworks. In the absence of definitional coherence, implementation practices may vary in scope, enforceability, and institutional accountability.

#### 3.1.2. Reactive Policy Enforcement and Responsibility Frameworks

This theme focuses on the procedural mechanics through which fragrance-free policies are operationalized. Many policies referenced complaint processes or occupational health review pathways, yet omitted progressive discipline, suggesting a predominantly reactive governance model rather than proactive environmental control. The allocation of burden of proof further illustrates this pattern. Several policies did not clearly specify whether responsibility rested with the institution or the individual. This distribution carries equity implications. Policies that rely on individualized complaint or medical verification processes may inadvertently disadvantage individuals with MCS or fragrance sensitivity by imposing administrative and evidentiary burdens. From a governance standpoint, unclear allocation of responsibility weakens regulatory coherence and may reduce perceived legitimacy. In environmental policy design, preventive approaches emphasize upstream source control, whereas complaint-driven models reflect downstream remediation. The predominance of reactive mechanisms observed across documents suggests that many fragrance-free policies function as accommodation tools rather than comprehensive environmental health controls.

#### 3.1.3. Policy Implementation Supports and Structural Integration

This theme examines the extent to which fragrance-free policies are operationalized through institutional support and embedded within organizational governance frameworks. Across the reviewed policies, implementation measures were unevenly distributed, revealing a pattern in which communication-based measures were more common than structural interventions. However, deeper institutional integration was less consistent. Fewer than half of the policies incorporated supply-chain or procurement controls or addressed facilities and maintenance systems. Training provisions were referenced in only a small proportion of documents, and formal review cycles were specified in just over one-fifth of policies. This distribution indicates that implementation frequently relied on communication-based tools rather than systemic environmental controls. While signage and policy visibility may contribute to awareness, they have limited capacity to address institutional sources of fragrance exposure. Reliance on voluntary compliance places greater emphasis on individual behavior than on environmental design. Inclusion of contractors and third-party providers was inconsistent across policies, indicating a potential exposure gap given their role in introducing fragranced products into institutional settings. Although explicit references to MCS were reported in several policies, detailed procedural guidance on accommodation was often limited or absent. Together, these findings suggest that many fragrance-free policies operate primarily at the level of normative communication rather than integrated environmental management. The limited incorporation of procurement standards, facilities practices, training, and evaluation mechanisms indicates partial institutionalization of fragrance governance. From an environmental health perspective, stronger structural implementation components may be necessary to align fragrance-free policies with established IAQ source-control principles and ensure consistent exposure reduction within shared indoor environments.

#### 3.1.4. Cross-Cutting Governance Gaps

Across the three themes, several cross-cutting governance gaps emerged. For example, the combination of unclear burden of proof, limited consequence structures for non-compliance, and infrequent monitoring or evaluation cycles constrains accountability. Although 74.6% of policies named a responsible authority, fewer than one-quarter specified monitoring or review mechanisms, limiting opportunities to assess implementation effectiveness. Accommodation pathways were inconsistently formalized, suggesting that access processes may remain informal or discretionary. Cultural exemptions were rare, and policy rationales rarely addressed broader social or environmental justice dimensions beyond references to MCS. The framing of fragrance exposure as either voluntary courtesy or individualized accommodation may limit recognition of broader environmental justice dimensions. Taken together, these gaps suggest that fragrance-free policies remain in a transitional governance phase: widely visible but unevenly embedded within organizational accountability systems. Without structural integration—such as procurement standards, contractor compliance, training, and evaluation—policies risk functioning symbolically rather than operationally.

## 4. Discussion

Although fragrance-free policies have been widely adopted, they demonstrate substantial heterogeneity in terminology, scope, enforceability, and operational strength. This variability in policy framing aligns with prior research highlighting the evolving and fragmented nature of fragrance exposure governance and the absence of standardized regulatory frameworks for managing fragrance product exposure in indoor environments [42]. Inconsistent operational definitions were a central finding of this review, suggesting that fragrance-free initiatives have developed in a decentralized manner, complicating implementation, comparability, and evaluation [45]. Reliance on voluntary compliance observed in this review remains common and consistent with previous literature [14], suggesting that policies that establish default fragrance-free environments or employ directive language more clearly articulate institutional responsibility and may offer more equitable and reliable protection. However, the inclusion of contractors and third-party providers was inconsistently addressed across policies, revealing a structural exposure gap given their role in procurement, cleaning, and facilities management, through which fragranced products could potentially be introduced into institutional settings.

Environmental health literature identifies fragranced consumer products as significant and under-regulated sources of indoor air contaminants [56], underscoring the need for coherent institutional governance. Enforcement mechanisms were predominantly reactive, relying on complaint-driven processes and often lacking clearly defined consequences for non-compliance or clear allocation of responsibility. This pattern mirrors broader occupational health and disability accommodation frameworks in which environmental protections depend on individual reporting rather than proactive risk management [57]. The burden of proof was frequently unclear or implicitly placed on affected individuals. Prior research has documented that individuals with MCS encounter institutional and policy-related barriers to accommodation and recognition [44]. Policies that explicitly assign institutional responsibility through directive language align more closely with public health principles of environmental control, primary prevention, and accessibility obligations recognized within human rights frameworks. These principles are consistent with accessibility obligations established under the United Nations Convention on the Rights of Persons with Disabilities (UN CRPD), which requires States Parties to identify and remove barriers in the built environment to ensure equal participation. Conversely, frameworks that require individuals to initiate accommodation processes may reduce accessibility and equity [53].

Implementation strategies were largely awareness-based, with comparatively few policies incorporating structural support such as procurement controls, contractor compliance, training mandates, or monitoring frameworks. Although responsible authorities were often identified, limited evaluation mechanisms constrained opportunities for accountability, continuous improvement, and evidence generation. Prior literature highlights that sustainability of workplace health interventions depends on governance structures, leadership accountability, and feedback loops [54]. Moreover, IAQ research consistently emphasizes source control as the primary and most effective exposure reduction strategy [58,59], suggesting that reliance on communication-based approaches alone is insufficient to meaningfully reduce fragrance-related exposures. Empirical IAQ monitoring data further demonstrates that environments implementing fragrance-free policies are associated with markedly lower VOC concentrations compared to non-policy environments, reinforcing the centrality of source control in institutional fragrance governance [60]. Alignment with established IAQ hierarchy-of-controls frameworks may therefore provide a structured pathway for strengthening institutional fragrance governance by prioritizing elimination and substitution strategies over behavioural compliance alone.

Complementary survey research further demonstrates that fragranced cleaning products, disinfectants, air fresheners, and laundry emissions are among the most frequently reported symptom triggers in workplace, healthcare, and residential environments [61]. In healthcare settings, indoor environments with chemical exposures have been associated with care avoidance and dissatisfaction, particularly where fragrance controls are absent [62]. Patterns of repeated accommodation denial across employment and housing contexts further indicate that reliance on complaint-based accommodation models may function as a structural barrier [63]. Together, these findings reinforce that fragrance governance must move beyond behavioural awareness approaches toward institutional-level source-control and accountability frameworks. Within this context, the compliance measures observed in this review primarily focused on identifying and removing fragranced exposure sources to mitigate related health risks. Prior research has emphasized the need for more robust reinforcement strategies to convey the importance of fragrance-free policies and support behaviour change [44]. Strengthening definitional clarity, institutional integration, and evaluation frameworks is therefore critical to advancing fragrance-free policies as durable environmental health interventions rather than reactive accommodation tools [42].

Based on the policy characteristics identified in this review, standardized definitions and compliance criteria may improve cross-sector consistency, while default fragrance-free standards could reduce reliance on individual complaints and better address institutional sources of exposure. Formalizing enforcement mechanisms, including defined roles, accountability structures, and proportional consequences, could enhance policy legitimacy while maintaining a supportive, educational approach. Integrating fragrance-free policies within organizational procurement systems, facilities operations, and training infrastructures is essential for sustained implementation. Structured staff training, alongside clear communication for visitors and the public, may strengthen awareness and compliance. Provision of appropriate accommodations for individuals with MCS or fragrance sensitivity may also promote equitable implementation. Finally, monitoring and evaluation mechanisms are necessary to assess effectiveness and refine policy governance over time. However, these recommendations are based on descriptive policy characteristics, and future research using robust empirical study designs is needed to evaluate the implementation, compliance, and health outcomes associated with fragrance-free policies.

### Strengths and Limitations

This review systematically mapped comprehensive policy characteristics across multiple sectors and jurisdictions. Structured data extraction enabled comparison of conceptual, procedural, and governance features, providing a comprehensive overview of current policy variability, as well as identifying structural gaps and priority areas for strengthening policy design. However, despite a comprehensive search of multiple academic databases and grey literature sources, some relevant policies may have been missed due to access restrictions, non-publication, or indexing constraints. Reliance on publicly available documents may not capture internal practices or informal enforcement mechanisms. Consequently, this review cannot draw conclusions regarding the effectiveness or broader impacts of fragrance-free policies. Lack of peer-reviewed studies also limits the generalizability of the findings and highlights an important gap in the literature. Findings from this review primarily reflect fragrance-free policy approaches within North American institutional and regulatory contexts and should not be assumed to represent practices in other jurisdictions. Additionally, heterogeneity in policy details limited direct cross-setting comparisons. Restricting inclusion to English- and French-language sources may have excluded relevant policies from other jurisdictions. Finally, as a scoping review, this study did not evaluate outcome effectiveness or causal impacts on IAQ or health indicators.

## 5. Conclusions

Fragrance-free policies represent a growing institutional strategy to address fragranced product exposure and support inclusive indoor environments. This review demonstrates that, although widely adopted, policies vary substantially in definitions, scope, enforcement mechanisms, and implementation supports. Many rely primarily on awareness and voluntary compliance, with fewer incorporating structural governance measures or evaluation frameworks. Strengthening definitional clarity, establishing default fragrance-free standards, integrating procurement and facilities systems, and formalizing accommodation and monitoring processes represent key opportunities to enhance policy effectiveness. Advancing these elements is essential to position fragrance-free policies as durable environmental health interventions that promote health, accessibility, and equity across institutional settings.

## Data Availability

No new data were created or analyzed in this study. Data sharing is not applicable to this article.

## Supplementary Materials

The following supporting information can be downloaded at: https://www.mdpi.com/article/10.3390/ijerph23081089/s1, File S1: PRISMA-ScR-Checklist; Table S1: Identification and Scope; Table S2: Substance and Mechanics; Table S3: Implementation and Equity.

## Abbreviations

The following abbreviations are used in this manuscript:

IAQ	Indoor Air Quality
MCS	Multiple Chemical Sensitivity
WHO	World Health Organization
IFRA	International Fragrance Association
AMA	American Medical Association
TRP	Transient Receptor Potential
TILT	Toxicant-Induced Loss of Tolerance
