# Supplementary material for: Fragrance-Free Policies: A Scoping Review of Definitions, Implementation, and Gaps
Source: Int J Environ Res Public Health. 2026 Aug 21;23(8):1089. doi: 10.3390/ijerph23081089 (PMC13513036; doi:10.3390/ijerph23081089)
Supplement: Supplementary file 1 [file ijerph-23-01089-s001.zip › Table S1.pdf]

Table S1: Identification and Scope

Record ID	Policy Title	Issuing Organization	City/ Region	Country	Setting	Policy status	Policy Tier	Year Adopted	Year Last Revised	Explicit MCS Reference	Applies to Visitors	URL
1.	Fragrance Regulation H-135.902	AMA	National	USA	Healthcare, workplace	Advisory	T2	2025	NR	Yes	No	https://www.ama-assn.org/system/files/a25-handbook-combined.pdf
2.	Scent-Free Policy for the Workplace	CCOHS	National	Canada	Workplace	Guideline	T5	NR	2024	Yes	Yes	https://www.ccohs.ca/oshanswers/hsprograms/scent_free.htm
3.	Scent-Awareness Guideline	Sheridan College	Oakville, ON	Canada	Education	Guideline	T5	NR	NR	Yes	Yes	https://media-www.sheridancollege.ca/-/media/project/sheridan/shared/files/student-life/campus-services/accessibility/scent-awareness-guidelines.pdf
4.	Scent Safety Guidelines	University of Victoria	Victoria, BC	Canada	Education	Guideline	T5	NR	NR	Yes	Yes	https://www.uvic.ca/ohse/_assets/docs/occupational-health/scent-safety-guidelines.pdf
5.	Scent Sensitive Workplace	City of Regina	Regina, SK	Canada	Workplace	Advisory	T2	2008	2015	No	Yes	https://www.local21.ca/fileadmin/local21/storage/City_of_Regina_Policies/Scent-Sensitive-Policy.pdf
6.	Scented Products Awareness Guidelines	Ontario Tech University	Oshawa, ON	Canada	Workplace, Education	Guideline	T5	NR	NR	Yes	Yes	https://shared.ontariotechu.ca/shared/department/healthandsafety/documents/uoit-scented-products-awareness-guidelines.pdf
7.	Scent Safety in the Workplace	WorkSafeBC	Vancouver, BC	Canada	Workplace	Advisory	T2	NR	NR	Yes	Yes	https://www.eha-ab.ca/acfp/docs/WorkSafeBCscentSafety.pdf
8.	Scent Free Workplace	Saskatchewan Cancer Agency	SK	Canada	Workplace	Mandatory	T1	2009	2016	No	Yes	https://saskcancer.ca/sites/default/files/2024-01/HR-506.pdf
9.	Scent Reduction Policy	Halifax Regional Municipality	Halifax, NS	Canada	Workplace	Mandatory	T1	1996	2012	Yes	Yes	https://cdn.halifax.ca/sites/default/files/documents/about-the-city/employment/scentreductionpolicy_final.pdf
10.	Scent-Free-Workplace-Policy	Nipissing First Nation	Garden Village, ON	Canada	Workplace	Mandatory	T1	2020	NR	Yes	Yes	https://nfn.ca/wp-content/uploads/2020/12/Scent-Free-Workplace-Policy.pdf
11.	Scent-Free Policy	Strong Start Charitable Organization	Waterloo, ON	Canada	Workplace	Advisory	T2	2025	NR	Yes	Yes	https://strongstart.ca/wp-content/uploads/Scent-Free_Workplace_Policy_Version_1_Web.pdf
12.	Scent-Free Initiative	University of Prince Edward Island	Charlottetown, PEI	Canada	Education, indoor, common spaces.	Voluntary/ advisory	T2	2007	NR	Yes	Yes	https://www.upei.ca/policy/adm/hrd/ohs/0004
13.	Scent-Free Policy	Fraser Health	Surrey, BC	Canada	Workplace, healthcare facilities	Mandatory	T1	2009	2022	Yes	Yes	https://www.fraserhealth.ca/-/media/Project/FraserHealth/FraserHealth/About-Us/Accountability/Policies/ScentedProducts-Policy-201802.pdf
14.	Scent Free Policy	Rideau Community Health Services	ON	Canada	Community Health Centre	Mandatory	T1	NR	NR	Yes	No	https://www.rideauchs.ca/client-relations/scent-free-policy
15.	Employment – Scent-Free Policy	Innisfil ideaLAB & Library	Innisfil, ON	Canada	Library facilities	Mandatory for staff, advisory for public	T1	2004	2022	Yes	Yes	https://www.innisfilidealab.ca/wp-content/uploads/2022/04/E-Scent-Free-Policy-E-2022-06.pdf
16.	Scent Free Environment	St. Joseph's Healthcare Hamilton (SJHH)	Hamilton, ON	Canada	Workplace, Healthcare	Mandatory	T1	2003	2019	No	Yes	https://www.stjoes.ca/patients-visitors/your-visit-or-stay/scent-free-campuses/007-ohs.pdf
17.	"Share the Air" Scent Free Policy	Town of Digby	NS	Canada	Workplace	Mandatory	T1	2023	NR	Yes	Yes	https://www.digby.ca/917-admin-policy-2023-01-scent-free-share-the-air-policy/file.html

18.	AOHC Scent Free Policy	Association of Ontario Health Centres	Toronto, ON	Canada	Healthcare, workplace	Mandatory	T1	NR	NR	Yes	Yes	https://www.allianceon.org/sites/default/files/documents/AOHC%20Scent%20Free%20Policy.pdf
19.	Scent Free Policy	Town of Niagara-on-the-Lake	ON	Canada	Public buildings	Mandatory	T1	2017	NR	Yes	Yes	https://domino.notl.com/sites/notl/NOTLCOTW.nsf/1D5DF412E5A087258525802F0055947B/FE42627DEE1E5EFF852581AF0066A45E
20.	Environmental sensitivities and scent-free policies	Canadian Human Rights Commission	National	Canada	Workplace	Guideline	T5	NR	2026	Yes	Yes	https://www.chrc-ccdp.gc.ca/resources/publications/environmental-sensitivities-and-scent-free-policies
21.	Indoor Air Quality: Scent Free Policy	Department of Health	Halifax, NS	Canada	Healthcare	Mandatory	T1	2000	NR	Yes	Yes	https://casle.ca/wp-content/uploads/2021/05/Dept-of-Health-Scent-Free-Policy-2000.pdf
22.	Scent Free Workplace	David Thompson Health Region	AB	Canada	Healthcare	Advisory	T2	2008	2008	No	Yes	https://www.eha-ab.ca/chemical/docs/DTHR%20Scent%20Free%20Work%20Place%20April%2008.pdf
23.	Scent safety	Eastern Health	NL	Canada	Healthcare	Mandatory	T1	2015	NR	Yes	Yes	https://adultrehab.easternhealth.ca/wp-content/uploads/sites/29/2020/02/HRScentSafetyPolicy-1.pdf
24.	Scent-free Workplace	City of Welland	ON	Canada	Workplace	Advisory	T2	NR	2020	No	Yes	https://www.welland.ca/bylaws/Policies/HR-004.pdf
25.	Scent-Free	University Health Network	Toronto, ON	Canada	Healthcare	Advisory	T2	NR	NR	No	Yes	https://www.uhn.ca/OurHospitals/Pages/smoke_scent_free_UHN.aspx
26.	PSAC Statement on Environmental Sensitivities	Public Service Alliance of Canada	National	Canada	Workplace	Advisory	T2	2017	2025	Yes	Yes	https://psacunion.ca/psac-statement-environmental-sensitivities
27.	Scented Product Program	York University	Toronto, ON	Canada	Education, workplace	Voluntary	T5	2015	NR	Yes	Yes	https://www.yorku.ca/
28.	Guidelines on the Use of Perfumes and Scented Products	University of Toronto	Toronto, ON	Canada	Education	Guideline	T5	NR	NR	Yes	Yes	https://ehs.utoronto.ca/our-services/occupational-hygiene-safety/guidelines-on-the-use-of-perfumes-and-scented-products/
29.	Scent Free Awareness	Alberta Health Services	AB	Canada	Workplace	Advisory	T2	NR	NR	Yes	No	https://www.eha-ab.ca/chemical/docs/AHSGuidingPractice.pdf#:~:text=Employees%20and%20others%20working%20on%20behalf%20of,or%20wearing%20scented%20items%20into%20our%20workplace.
30.	Scent Free Environment	Royal Ottawa Health Care Group	Ottawa, ON	Canada	Workplace	Voluntary	T5	2012	2018	No	Yes	https://www.theroyal.ca/wp-content/uploads/2025/10/CORP-IV-i-240-Scent-Free-rev.-SS-Feb-8-2022.pdf
31.	Scent Awareness Policy	Simcoe County District School Board	Midhurst, ON	Canada	Education, workplace	Voluntary	T5	2008	NR	Yes	Yes	https://www.simcoedeces.com/uploads/1527186929_Scent%20Awareness%20Policy.pdf
32.	Scent Policy	Government of Newfoundland and Labrador – Treasury Board Secretariat	NL	Canada	Workplaces	Mandatory	T1	NR	NR	No	Yes	https://www.gov.nl.ca/exec/tbs/policies/healthy-workplace/scent/#:~:text=Scented%20products%20are%20to%20be,scented%20products%20in%20the%20workplace.
33.	Scent-Free Policy	Schulich School of Medicine & Dentistry	London, ON	Canada	Education, healthcare, workplace	Voluntary	T5	NR	NR	No	No	https://www.schulich.uwo.ca/humanresources/leader_administrator_tools/scentfree_policy.html
34.	Scents in the Workplace	University of British Columbia	Kelowna, BC	Canada	Education	Voluntary	T5	NR	NR	Yes	No	https://operations.ok.ubc.ca/health-and-safety/environmental-health/scents-in-the-workplace/

35.	Scent-Free Workplace Policy	Courts Division, Manitoba Justice	MB	Canada	Workplace, public spaces	Advisory	T2	NR	NR	Yes	Yes	https://www.manitobacourts.mb.ca/site/assets/files/1129/scentfree_workplace.pdf
36.	Scent Free Guideline	University of Winnipeg	Winnipeg, MB	Canada	Education	Guideline	T5	NR	NR	Yes	Yes	https://www.uwinnipeg.ca/safety/docs/scent-free-guideline-booklet.pdf
37.	Scent-Free Program	Memorial University	NL	Canada	Education	Mandatory	T1	NR	NR	Yes	Yes	https://www.mun.ca/medicine/faculty-and-staff-resources/administrative-services/health-and-safety/scent-free-program/
38.	2024 Accessibility Progress Report	Library of Parliament	Ottawa, ON	Canada	Workplace	Advisory	T3	NR	NR	Yes	No	https://lop.parl.ca/staticfiles/PublicWebsite/Home/About/Accessibility/2024_progress_report-e_final_LARGE_PRINT.pdf
39.	Fragrance-Free Environment	United States Access Board	Washington, D.C.	USA	Public spaces	Advisory	T2	NR	NR	Yes	No	https://www.access-board.gov/about/policy/ffe.html
40.	Scents	Canadian Lung Association	National	Canada	Workplace, home, public spaces	Guideline	T5	NR	NR	Yes	No	https://www.lung.ca/lung-health/scents
41.	Fragrance Sensitivity	Job Accommodation Network	National	USA	Workplace	Guideline	T5	NR	NR	Yes	No	https://askjan.org/disabilities/Fragrance-Sensitivity.cfm
42.	Becoming Scent Free	Breakspear Medical	Hemel Hempstead, Hertfordshire	UK	Healthcare	Advisory	T2	NR	NR	Yes	Yes	https://www.breakspearmedical.com/patient-information/becoming-scent-free/
43.	Scent Free Environment	Vancouver Island North	BC	Canada	Education, workplace	Mandatory	T1	2011	NR	Yes	Yes	https://www.sd85.bc.ca/wordpress/wp-content/uploads/2019/07/5-35.pdf
44.	Scent Free Environment for Union Function	British Columbia Government Employee's Union	BC	Canada	Workplace	Advisory	T2	2001	NR	Yes	No	https://d3n8a8pro7vhmx.cloudfront.net/bcgeu/pages/5066/attachments/original/1505236941/Scent_Free_Environment.pdf?1505236941
45.	Occupational Health & Safety – Scent Free Environment	Western Health	NL	Canada	Healthcare, workplace	Mandatory	T1	2008	2017	No	Yes	https://westernhealth.nl.ca/uploads/Policies/scent-free.pdf
46.	Scent-Free Policy	Newfoundland and Labrador Public Sector Pensioners' Association	NL	Canada	Workplace	Advisory	T2	NR	NR	No	Yes	https://nlpspa.ca/wp-content/uploads/2022/04/NLPSPA-Scent-Free-Policy.pdf
47.	Un air sain et sans produits parfumés	City of Gatineau	QC	Canada	Workplace, public spaces	Advisory	T2	NR	NR	Yes	No	https://www.gatineau.ca/portail/default.aspx?p=guichet_municipal/communaute/syndrome_sensibilite_chimique_multiple
48.	Indoor Environmental Quality Policy	CDC	National	USA	Workplace	Advisory	T2	NR	NR	Yes	Yes	US Centers for Disease Control Follows Fragrance-Free Policy - Invisible Disabilities® Association
49.	Scent Reduction Policy	Town of Truro	NS	Canada	Workplace	Advisory	T2	NR	NR	Yes	No	file
50.	Fragrance Free Policy	Oregon Police Department	Oregon	USA	Workplace	Mandatory	T1	NR	NR	No	No	OREGON POLICE DEPARTMENT Records Division.pdf - Google Drive
51.	Fragrance-Free Policy	Governor's Council on Disability	Missouri	USA	Workplace	Voluntary	T5	NR	NR	Yes	Yes	GCD Fragrance Free Policy
52.	Fragrance Free Environment-Sample Policy	Ottawa Public Health	ON	Canada	Workplace	Template	T5	NR	NR	Yes	Yes	Fragrance Free Environment – Sample Policy
53.	Fragrance-Free Policy	National Federation of the Blind of Maryland	Baltimore	USA	Workplace	Mandatory	T1	2017	NR	Yes	No	https://nfbmd.org/fragrance-free-policy
54.	Fragrance Free/ Chemical Free Policy	Alaska Healing Touch	Alaska	USA	Healthcare	Advisory	T2	NR	NR	Yes	No	https://www.alaskahealingtouch.com/fragrance-free-policy
55.	Fragrance-Free Workplace	Department of Corrections	Montana	USA	Workplace	Advisory	T2	2010	2015	Yes	Yes	https://cor.mt.gov/DataStatsContractsPoliciesProcedures/DataDocumentsandLinks/DOCPolicies/Chapter1/1.3.42-Fragrance-Free-Workplace-06.01.pdf

56.	Fragrance Policy	Santa Fe Vipassana Sangha	New Mexico	USA	Public space	Advisory	T2	NR	NR	Yes	No	https://santafevipassana.org/fragrance-policy/
57.	Fragrance Policy	Shaughnessy Heights United Church	Vancouver, BC	Canada	Public space	Mandatory	T1	NR	NR	Yes	Yes	https://www.shuc.ca/policies-procedures/
58.	Fragrance Free Policy	Gay City	Seattle	USA	Public spaces	Mandatory	T1	NR	NR	No	No	https://www.gaycity.org/accessibility/
59.	Fragrances and Scented Products in the Workplace	Corporate Health and Safety Program	London, ON	Canada	Workplace	Mandatory	T1	2017	2023	Yes	Yes	https://london.ca/sites/default/files/2024-04/OHS%204.4-12%20Fragrances%20and%20Scented%20Products%20in%20the%20Workplace.pdf
60.	Did you know fragrances can make people ill	Mast Cell Action	National	UK	Workplace, healthcare, public spaces	Awareness poster	T4	NR	NR	No	No	https://www.mastcellaction.org/assets/_/2026/01/07/0bee03a6-440d-4e84-9c0d-6019ad46fb69/did-you-know-fragrances-poster.pdf?v=1
61.	Fragrance-Free Workplace Policy	Manatal	National	Singapore, Thailand	Workplace	Advisory	T2	NR	NR	Yes	Yes	https://www.manatal.com/glossary/fragrance-free-workplace-policy
62.	The smell is bothering some people (please be considerate of the smell)	Osaka Prefectural Government	Osaka City	Japan	Workplace, healthcare, public spaces	Awareness poster	T4	NR	NR	Yes	No	https://www.pref.osaka.lg.jp/o100040/chikikansen/kaorihairyo/index.html
63.	Non Fragrance Week	Swedish Asthma and Allergy Association	National	Sweden	Public places	Awareness campaign	T4	NR	NR	No	No	https://www.efanet.org/news/3253-no-perfume-in-public-places-please-say-the-swedes

Note: NR = Not Reported in source document; Policy tiers were assigned according to the predominant purpose, level of authority, and policy characteristics of each record. Where a document contained characteristics of multiple tiers, classification was based on the dominant policy approach, with mandatory and enforceable policy elements taking precedence over educational or awareness-oriented components. Tier 1 (enforceable institutional policies with mandatory language), Tier 2 (advisory institutional policies), Tier 3 (government directives or accessibility plan), Tier 4 (awareness campaigns such as posters or educational materials), and Tier 5 (toolkits, templates, or advocacy resources).
