# Supplementary material for: Fragrance-Free Policies: A Scoping Review of Definitions, Implementation, and Gaps
Source: Int J Environ Res Public Health. 2026 Aug 21;23(8):1089. doi: 10.3390/ijerph23081089 (PMC13513036; doi:10.3390/ijerph23081089)
Supplement: Supplementary file 1 [file ijerph-23-01089-s001.zip › Table S2.pdf]

Table S2: Substance and Mechanics

Record ID	Definition Provided	Default Fragrance-Free Standard	Complaint Mechanism Specified	Enforcement Mechanisms Described	Consequences for Non-Compliance	Burden of Proof
1.	Yes	Yes	No	Yes	No	Unclear
2.	Yes	Yes	Yes	Yes	No	Unclear
3.	No	No	Yes	Yes	No	Individual
4.	No	No	Yes	Yes	No	Individual
5.	Yes	No	Yes	Yes	No	Individual
6.	No	No	Yes	Yes	No	Unclear
7.	No	No	Yes	Yes	No	Unclear
8.	No	Yes	No	No	No	Employer
9.	Yes	Yes	Yes	Yes	No	Employer
10.	Yes	Yes	Yes	Yes	No	Employer
11.	No	No	Yes	Yes	Yes	Individual
12.	Yes	No	No	Yes	No	Unclear
13.	Yes	Yes	Yes	Yes	No	Employer
14.	Yes	Yes	Yes	Yes	Yes	Employer
15.	Yes	Yes	Yes	Yes	No	Employer
16.	Yes	Yes	Yes	Yes	No	Employer
17.	Yes	Yes	Yes	Yes	Yes	Employer
18.	Yes	Yes	Yes	Yes	No	Employer
19.	Yes	Yes	Yes	No	Yes	Employer
20.	Yes	No	Yes	Yes	Yes	Unclear
21.	Yes	Yes	Yes	Yes	No	Employer
22.	Yes	No	Yes	Yes	Yes	Unclear
23.	Yes	Yes	Yes	Yes	Yes	Employer
24.	Yes	No	Yes	Yes	Yes	Individual
25.	No	No	Yes	No	No	Individual
26.	No	No	Yes	No	No	Unclear
27.	Yes	No	Yes	Yes	Yes	Unclear
28.	No	No	Yes	Yes	No	Individual
29.	No	No	No	No	No	Unclear
30.	Yes	No	Yes	Yes	Yes	Unclear
31.	No	No	Yes	Yes	No	Individual
32.	Yes	Yes	No	No	No	Employer
33.	No	No	No	No	No	Unclear
34.	Yes	No	Yes	Yes	No	Unclear
35.	No	No	No	No	No	Unclear
36.	Yes	No	Yes	Yes	No	Unclear
37.	Yes	Yes	Yes	No	No	Employer
38.	No	No	Yes	Yes	No	Unclear
39.	No	No	No	Yes	No	Unclear
40.	Yes	No	No	Yes	No	Unclear
41.	Yes	No	No	No	No	Unclear
42.	No	No	No	No	No	Unclear
43.	No	Yes	Yes	Yes	No	Employer
44.	No	No	Yes	Yes	No	Unclear
45.	Yes	Yes	Yes	Yes	Yes	Employer
46.	Yes	No	No	No	No	Unclear
47.	No	No	No	No	No	Unclear
48.	No	No	No	No	No	Unclear
49.	No	No	Yes	Yes	No	Individual
50.	No	Yes	No	No	Yes	Employer
51.	No	No	No	Yes	No	Unclear
52.	No	No	No	Yes	Yes	Unclear

53.	No	Yes	No	No	No	Employer
54.	No	No	No	No	No	Unclear
55.	No	No	Yes	Yes	No	Individual
56.	No	No	No	No	No	NA
57.	No	Yes	No	Yes	No	NA
58.	Yes	Yes	No	No	No	NA
59.	Yes	Yes	No	Yes	No	Employer
60.	No	No	No	No	No	NA
61.	No	No	Yes	Yes	Yes	Individual
62.	No	No	No	No	No	NA
63.	No	No	No	No	No	NA

Note: NA = Not Applicable to policy type; Burden of Proof = Defined by locus of responsibility for compliance—employer (default fragrance-free environment), individual (triggered by request or documentation), or unclear (non-specific or advisory language).
