# Supplementary material for: Fragrance-Free Policies: A Scoping Review of Definitions, Implementation, and Gaps
Source: Int J Environ Res Public Health. 2026 Aug 21;23(8):1089. doi: 10.3390/ijerph23081089 (PMC13513036; doi:10.3390/ijerph23081089)
Supplement: Supplementary file 1 [file ijerph-23-01089-s001.zip › Table S3.pdf]

Table S3: Implementation and Equity

Record ID	Medical Documentation Required	Cultural Exemptions	Supply Chain Control	Facilities/ Maintenance Included	Training Referenced	Signage Required	Publicly Posted	Review Cycle Specified	Responsible Authority Named	Applies to Contractor/Third Parties	Policy Trigger/ Rationale Stated
1.	No	No	No	No	No	No	Yes	No	No	Yes	Yes
2.	No	No	Yes	Yes	Yes	Yes	Yes	No	Yes	Yes	Yes
3.	No	No	Yes	Yes	No	No	Yes	No	Yes	No	Yes
4.	No	No	Yes	Yes	No	Yes	Yes	No	Yes	No	Yes
5.	Yes	No	Yes	Yes	No	No	Yes	Yes	Yes	Yes	Yes
6.	No	No	No	No	No	Yes	Yes	No	Yes	Yes	Yes
7.	No	No	Yes	Yes	Yes	Yes	Yes	Yes	Yes	Yes	Yes
8.	No	No	No	No	No	No	Yes	No	Yes	Yes	Yes
9.	No	No	No	No	No	Yes	Yes	Yes	Yes	No	Yes
10.	No	Yes	Yes	Yes	No	Yes	Yes	No	Yes	Yes	Yes
11.	No	No	Yes	Yes	Yes	Yes	Yes	No	Yes	No	Yes
12.	No	No	Yes	No	No	Yes	Yes	No	No	No	Yes
13.	Yes	Yes	Yes	Yes	No	No	Yes	Yes	Yes	Yes	Yes
14.	No	No	No	Yes	No	Yes	Yes	No	Yes	Yes	Yes
15.	No	No	Yes	Yes	No	Yes	Yes	Yes	Yes	No	Yes
16.	No	No	Yes	Yes	No	Yes	Yes	Yes	Yes	Yes	Yes
17.	No	No	Yes	Yes	No	Yes	Yes	Yes	Yes	Yes	Yes
18.	No	No	Yes	Yes	No	Yes	Yes	No	No	No	Yes
19.	No	No	Yes	Yes	Yes	Yes	Yes	No	Yes	No	Yes
20.	No	No	Yes	No	No	Yes	Yes	No	Yes	Yes	Yes
21.	No	No	Yes	Yes	No	Yes	Yes	No	Yes	No	Yes
22.	No	No	Yes	Yes	No	Yes	Yes	Yes	Yes	Yes	Yes
23.	Yes	No	Yes	Yes	No	Yes	Yes	Yes	Yes	Yes	Yes
24.	No	No	Yes	Yes	No	Yes	Yes	No	Yes	Yes	Yes
25.	No	No	Yes	No	No	No	Yes	No	No	No	No
26.	No	No	No	No	No	No	Yes	No	No	No	Yes
27.	Yes	No	No	Yes	Yes	Yes	Yes	Yes	Yes	Yes	Yes
28.	No	No	No	Yes	No	Yes	Yes	Yes	Yes	No	Yes
29.	No	No	No	No	No	Yes	Yes	No	No	No	Yes
30.	No	No	Yes	Yes	No	Yes	Yes	No	Yes	Yes	Yes
31.	No	No	No	No	Yes	Yes	Yes	No	Yes	No	Yes
32.	No	No	Yes	Yes	No	Yes	Yes	No	Yes	No	Yes
33.	No	No	No	No	No	No	Yes	No	Yes	No	Yes
34.	No	No	No	No	No	No	Yes	No	Yes	No	Yes
35.	No	No	No	No	No	No	Yes	No	Yes	Yes	Yes
36.	No	No	No	No	No	No	Yes	No	Yes	No	Yes
37.	No	No	Yes	Yes	No	Yes	Yes	No	Yes	No	Yes
38.	No	No	Yes	Yes	Yes	Yes	Yes	No	Yes	No	Yes
39.	No	No	No	No	No	Yes	Yes	No	Yes	No	Yes
40.	No	No	No	No	No	Yes	Yes	No	Yes	No	Yes
41.	No	No	No	No	No	No	Yes	No	Yes	No	Yes
42.	No	No	No	No	No	No	Yes	No	No	No	Yes
43.	No	No	No	No	Yes	Yes	Yes	No	Yes	No	Yes
44.	No	No	No	No	No	No	Yes	No	Yes	No	Yes
45.	No	No	Yes	Yes	No	Yes	Yes	Yes	Yes	Yes	Yes
46.	No	No	Yes	Yes	No	Yes	Yes	No	Yes	Yes	Yes
47.	No	No	No	No	No	No	Yes	No	No	No	Yes
48.	No	No	Yes	Yes	No	No	Yes	No	Yes	Yes	Yes
49.	No	No	No	Yes	No	Yes	Yes	No	Yes	No	Yes
50.	No	No	No	No	No	No	Yes	No	No	No	Yes
51.	No	No	Yes	Yes	No	Yes	Yes	No	Yes	Yes	Yes

52.	No	No	No	No	No	Yes	Yes	No	Yes	No	Yes
53.	No	No	No	No	No	No	Yes	No	No	No	Yes
54.	No	No	No	No	No	No	Yes	No	No	No	Yes
55.	No	No	No	No	No	No	Yes	No	Yes	Yes	Yes
56.	No	No	No	No	No	No	Yes	No	No	No	Yes
57.	No	No	Yes	No	No	No	Yes	No	Yes	Yes	Yes
58.	No	No	No	No	No	No	Yes	No	No	Yes	Yes
59.	No	No	No	Yes	Yes	Yes	Yes	Yes	Yes	No	Yes
60.	No	No	No	No	No	No	Yes	No	No	No	NA
61.	No	No	No	No	Yes	No	Yes	Yes	Yes	Yes	Yes
62.	No	No	No	No	No	No	Yes	No	No	No	NA
63.	No	No	No	No	No	No	Yes	No	No	No	NA

Note: NA = Not Applicable to policy type
